# Supplementary material for: Parental opioid prescriptions and the risk of opioid use in adolescents and young adults: The HUNT Study linked with prescription registry data
Source: PLoS Med. 2025 Oct 23;22(10):e1004763. doi: 10.1371/journal.pmed.1004763 (PMC12548922; doi:10.1371/journal.pmed.1004763)
Supplement: S1 Table — (DOCX) [file pmed.1004763.s001.docx]

Table S1. Survey used for parental chronic MSK pain and body mass index variables

|  | Mothers | Fathers |
| --- | --- | --- |
| Parental chronic MSK pain variable |  |  |
| Same survey as offspring | 12,555 (68.2%) | 9,320 (59.2%) |
| Earlier survey than offspring | 5,327 (29.1%) | 5,915 (37.6%) |
| Later survey than offspring | 513 (2.8%) | 511 (3.2%) |
| Parental body mass index variable |  |  |
| Same survey as offspring | 15,613 (78.6%) | 12,533 (70.2%) |
| Earlier survey than offspring | 3,870 (19.5%) | 4,879 (27.8%) |
| Later survey than offspring | 401 (2.1%) | 395 (2.2%) |

MSK: musculoskeletal
