# Supplementary material for: Parental opioid prescriptions and the risk of opioid use in adolescents and young adults: The HUNT Study linked with prescription registry data
Source: PLoS Med. 2025 Oct 23;22(10):e1004763. doi: 10.1371/journal.pmed.1004763 (PMC12548922; doi:10.1371/journal.pmed.1004763)
Supplement: S2 Table — (DOCX) [file pmed.1004763.s002.docx]

Table S2. Parental age and opioid prescription stratified by missingness in parental chronic musculoskeletal pain variable

|  | Not missing pain | Missing pain |
| --- | --- | --- |
| Mother age, mean (SD) | 46.4 (6.2) | 43.9 (6.8) |
| Mother opioid prescriptions, n (%)  0  1  ≥2 | 15,413 (84.0)  1,559 (8.5)  1,367 (7.5) | 1,657 (86.6)  124 (6.5)  132 (6.9) |
| Father age, mean (SD) | 49.7 (6.7) | 47.9 (7.5) |
| Father opioid prescriptions, n (%)  0  1  ≥2 | 13,432 (85.8)  1,283 (8.2)  935 (6.0) | 2,247 (90.5)  125 (5.0)  110 (4.4) |

SD: standard deviation
