# Supplementary material for: Parental opioid prescriptions and the risk of opioid use in adolescents and young adults: The HUNT Study linked with prescription registry data
Source: PLoS Med. 2025 Oct 23;22(10):e1004763. doi: 10.1371/journal.pmed.1004763 (PMC12548922; doi:10.1371/journal.pmed.1004763)
Supplement: S3 Table — (DOCX) [file pmed.1004763.s003.docx]

Table S3. Effect of parental opioid prescription on risk of any opioid prescription in offspring for the full sample and restricted sample with information on both parents (i.e. trios)

|  | Full sample | | | |  | Restricted sample^a^ | | | |
| --- | --- | --- | --- | --- | --- | --- | --- | --- | --- |
| Any parental prescription | Person years | No. of cases | Crude,  HR (95% CI) | Adjusted^b^,  HR (95% CI) |  | Person years | No. of cases | Crude,  HR (95% CI) | Adjusted^b^,  HR (95% CI) |
| Mother |  |  |  |  |  |  |  |  |  |
| No | 94,066 | 3,975 | 1.00 (reference) | 1.00 (reference) |  | 80,082 | 3,334 | 1.00 (reference) | 1.00 (reference) |
| Yes | 16,812 | 870 | 1.25 (1.16-1.35) | 1.22 (1.14-1.32) |  | 13,923 | 701 | 1.24 (1.14-1.34) | 1.22 (1.12-1.32) |
| Father |  |  |  |  |  |  |  |  |  |
| No | 85,602 | 3,662 | 1.00 (reference) | 1.00 (reference) |  | 80,386 | 3,388 | 1.00 (reference) | 1.00 (reference) |
| Yes | 13,047 | 615 | 1.13 (1.04-1.23) | 1.11 (1.02-1.21) |  | 12,255 | 577 | 1.14 (1.05-1.25) | 1.12 (1.03-1.22) |

HR, hazard ratio; CI, confidence interval

^a^ Restricted sample of offspring where information from both parents were available

^b^ Adjusted for parental age at time offspring participated in HUNT survey (continuous), parental highest education (<12, ≥12 years), parental body mass index (continuous), offspring age (continuous) and survey of offspring participation (Young-HUNT3/HUNT3, Young-HUNT4/HUNT4)
