# Supplementary material for: Parental opioid prescriptions and the risk of opioid use in adolescents and young adults: The HUNT Study linked with prescription registry data
Source: PLoS Med. 2025 Oct 23;22(10):e1004763. doi: 10.1371/journal.pmed.1004763 (PMC12548922; doi:10.1371/journal.pmed.1004763)
Supplement: S4 Table — (DOCX) [file pmed.1004763.s004.docx]

Table S4. Effect of parental opioid prescription on risk of persistent opioid prescription in offspring for the full sample and restricted sample with information on both parents (i.e. trios)

|  | Full sample | | | |  | Restricted sample^a^ | | | |
| --- | --- | --- | --- | --- | --- | --- | --- | --- | --- |
| Any parental prescription | Person years | No. of cases | Crude,  HR (95% CI) | Adjusted^b^,  HR (95% CI) |  | Person years | No. of cases | Crude,  HR (95% CI) | Adjusted^b^,  HR (95% CI) |
| Mother |  |  |  |  |  |  |  |  |  |
| No | 95,413 | 183 | 1.00 (reference) | 1.00 (reference) |  | 81,185 | 146 | 1.00 (reference) | 1.00 (reference) |
| Yes | 17,127 | 58 | 1.83 (1.36-2.45) | 1.72 (1.28-2.31) |  | 14,166 | 45 | 1.82 (1.30-2.55) | 1.73 (1.24-2.43) |
| Father |  |  |  |  |  |  |  |  |  |
| No | 86,831 | 168 | 1.00 (reference) | 1.00 (reference) |  | 81,482 | 155 | 1.00 (reference) | 1.00 (reference) |
| Yes | 13,279 | 38 | 1.42 (0.99-2.03) | 1.36 (0.95-1.95) |  | 12,486 | 32 | 1.39 (0.95-2.03) | 1.33 (0.91-1.95) |

HR, hazard ratio; CI, confidence interval

^a^ Restricted sample of offspring where information from both parents were available

^b^ Adjusted for parental age at time offspring participated in HUNT survey (continuous), parental highest education (<12, ≥12 years), parental body mass index (continuous), offspring age (continuous) and survey of offspring participation (Young-HUNT3/HUNT3, Young-HUNT4/HUNT4)
