# Supplementary material for: Parental opioid prescriptions and the risk of opioid use in adolescents and young adults: The HUNT Study linked with prescription registry data
Source: PLoS Med. 2025 Oct 23;22(10):e1004763. doi: 10.1371/journal.pmed.1004763 (PMC12548922; doi:10.1371/journal.pmed.1004763)
Supplement: S5 Table — (DOCX) [file pmed.1004763.s005.docx]

Table S5. Effect of parental number of opioid prescriptions on risk of any opioid prescription in offspring stratified by offspring age

|  | <20 years | | | |  | ≥20 years | | | |
| --- | --- | --- | --- | --- | --- | --- | --- | --- | --- |
| No. of prescriptions | Person years | No. of cases | Crude,  HR | Adjusted^a^,  HR (95% CI) |  | Person years | No. of cases | Crude,  HR | Adjusted^a^,  HR (95% CI) |
| Mother |  |  |  |  |  |  |  |  |  |
| 0 | 66,830 | 2,362 | 1.00 (reference) | 1.00 (reference) |  | 27,237 | 1,613 | 1.00 (reference) | 1.00 (reference) |
| 1 | 6,661 | 262 | 1.14 (1.00-1.29) | 1.12 (0.99-1.27) |  | 2,553 | 166 | 1.10 (0.94-1.29) | 1.11 (0.94-1.30) |
| ≥2 | 5,505 | 272 | 1.43 (1.26-1.62) | 1.34 (1.18-1.52) |  | 2,093 | 170 | 1.37 (1.17-1.60) | 1.34 (1.14-1.57) |
| Father |  |  |  |  |  |  |  |  |  |
| 0 | 60,207 | 2,193 | 1.00 (reference) | 1.00 (reference) |  | 25,395 | 1,469 | 1.00 (reference) | 1.00 (reference) |
| 1 | 5,649 | 198 | 0.98 (0.84-1.13) | 0.96 (0.83-1.11) |  | 2,099 | 142 | 1.17 (0.98-1.39) | 1.17 (0.98-1.39) |
| ≥2 | 3,728 | 155 | 1.17 (1.00-1.38) | 1.12 (0.95-1.32) |  | 1,572 | 120 | 1.32 (1.10-1.59) | 1.29 (1.07-1.55) |

HR, hazard ratio; CI, confidence interval

^a^ Adjusted for parental age at time offspring participated in HUNT survey (continuous), parental highest education (<12, ≥12 years), parental body mass index (continuous), offspring age (continuous) and survey of offspring participation (Young-HUNT3/HUNT3, Young-HUNT4/HUNT4)
