# Supplementary material for: Parental opioid prescriptions and the risk of opioid use in adolescents and young adults: The HUNT Study linked with prescription registry data
Source: PLoS Med. 2025 Oct 23;22(10):e1004763. doi: 10.1371/journal.pmed.1004763 (PMC12548922; doi:10.1371/journal.pmed.1004763)
Supplement: S6 Table — (DOCX) [file pmed.1004763.s006.docx]

Table S6. Joint effect analysis restricted to offspring whose parents have information about chronic musculoskeletal pain at the same survey

|  | No opioid prescriptions | | | |  | ≥2 opioid prescriptions | | | |  | Within musculoskeletal pain strata estimates |
| --- | --- | --- | --- | --- | --- | --- | --- | --- | --- | --- | --- |
| Parental chronic MSK pain | Person years | No. of cases | Crude,  HR (95% CI) | Adjusted^a^,  HR (95% CI) |  | Person years | No. of cases | Crude,  HR (95% CI) | Adjusted^a^,  HR (95% CI) |  | Adjusted^a^,  HR (95% CI) |
| Mothers |  |  |  |  |  |  |  |  |  |  |  |
| No | 27,323 | 1,045 | 1.00 (reference) | 1.00 (reference) |  | 1,249 | 55 | 1.19 (0.91-1.56) | 1.14 (0.87-1.50) |  | 1.16 (0.88-1.52) |
| Yes | 29,506 | 1,357 | 1.16 (1.07-1.26) | 1.14 (1.05-1.23) |  | 4,277 | 247 | 1.52 (1.32-1.75) | 1.45 (1.26-1.67) |  | 1.27 (1.11-1.46) |
| Fathers |  |  |  |  |  |  |  |  |  |  |  |
| No | 23,991 | 951 | 1.00 (reference) | 1.00 (reference) |  | 1,117 | 57 | 1.32 (1.01-1.73) | 1.29 (0.99-1.68) |  | 1.28 (0.98-1.67) |
| Yes | 19,250 | 817 | 1.05 (0.95-1.15) | 1.03 (0.94-1.13) |  | 2,302 | 122 | 1.33 (1.10-1.60) | 1.29 (1.06-1.56) |  | 1.25 (1.03-1.51) |

MSK, musculoskeletal; HR, hazard ratio; CI, confidence interval

^a^ Adjusted for parental age at time offspring participated in HUNT survey (continuous), parental highest education (<12, ≥12 years), parental body mass index (continuous), offspring age (continuous) and survey of offspring participation (Young-HUNT3/HUNT3, Young-HUNT4/HUNT4)
