# Supplementary material for: Parental opioid prescriptions and the risk of opioid use in adolescents and young adults: The HUNT Study linked with prescription registry data
Source: PLoS Med. 2025 Oct 23;22(10):e1004763. doi: 10.1371/journal.pmed.1004763 (PMC12548922; doi:10.1371/journal.pmed.1004763)
Supplement: S7 Table — (DOCX) [file pmed.1004763.s007.docx]

Table S7. Number of parental opioid prescriptions and risk of opioid prescriptions in offspring removing those who had opioid prescription 1-year prior to participation

|  | Any opioid prescription | | | |  | Persistent opioid prescriptions | | | |
| --- | --- | --- | --- | --- | --- | --- | --- | --- | --- |
| No. of parental prescriptions | Person years | No. of cases | Crude  HR (95% CI) | Adjusted^a^,  HR (95% CI) |  | Person years | No. of cases | Crude  HR (95% CI) | Adjusted^a^,  HR (95% CI) |
| Mothers |  |  |  |  |  |  |  |  |  |
| 0 | 91,749 | 3,671 | 1.00 (reference) | 1.00 (reference) |  | 92,927 | 129 | 1.00 (reference) | 1.00 (reference) |
| 1 | 9,046 | 399 | 1.12 (1.01-1.25) | 1.13 (1.02-1.25) |  | 9,177 | 10 | 0.81 (0.43-1.54) | 0.84 (0.44-1.59) |
| ≥2 | 7,372 | 397 | 1.37 (1.24-1.52) | 1.33 (1.20-1.47) |  | 7,511 | 30 | 2.98 (2.00-4.43) | 2.94 (1.97-4.39) |
| Fathers |  |  |  |  |  |  |  |  |  |
| 0 | 83,542 | 3,397 | 1.00 (reference) | 1.00 (reference) |  | 86,934 | 168 | 1.00 (reference) | 1.00 (reference) |
| 1 | 7,522 | 316 | 1.05 (0.94-1.18) | 1.05 (0.93-1.17) |  | 7,857 | 12 | 0.81 (0.45-1.46) | 0.82 (0.45-1.47) |
| ≥2 | 5,127 | 244 | 1.19 (1.05-1.36) | 1.16 (1.02-1.32) |  | 5,412 | 26 | 2.51 (1.66-3.80) | 2.35 (1.55-3.57) |

HR, hazard ratio; CI, confidence interval

^a^ Adjusted for parental age at time offspring participated in HUNT survey (continuous), parental highest education (<12, ≥12 years), parental body mass index (continuous), offspring age (continuous) and survey of offspring participation (Young-HUNT3/HUNT3, Young-HUNT4/HUNT4)
