# Supplementary material for: Parental opioid prescriptions and the risk of opioid use in adolescents and young adults: The HUNT Study linked with prescription registry data
Source: PLoS Med. 2025 Oct 23;22(10):e1004763. doi: 10.1371/journal.pmed.1004763 (PMC12548922; doi:10.1371/journal.pmed.1004763)
Supplement: S8 Table — (DOCX) [file pmed.1004763.s008.docx]

Table S8. Descriptive characteristics of the sample stratified by number of opioid prescriptions in their mothers and fathers

|  | Mothers’ opioid prescriptions | | |  | Fathers’ opioid prescriptions | | |
| --- | --- | --- | --- | --- | --- | --- | --- |
| Characteristic | 0 | 1 | ≥2 |  | 0 | 1 | ≥2 |
| No. of adolescents/young adults | 16,505 | 1,673 | 1,480 |  | 14,980 | 1,402 | 1,018 |
| Age, mean (SD), years | 18.0 (4.3) | 17.8 (4.4) | 18.0 (4.4) |  | 18.1 (4.4) | 17.9 (4.4) | 18.2 (4.5) |
| Females, n (%) | 8,705 (52.7) | 883 (52.8) | 792 (53.5) |  | 7,802 (52.1) | 739 (52.7) | 563 (55.3) |
| Parental age, mean (SD), years | 46.3 (6.1) | 45.9 (6.5) | 45.8 (6.6) |  | 49.4 (6.5) | 49.2 (6.6) | 50.2 (7.0) |
| Parental higher education^a^, n (%) | 6,480 (39.3) | 625 (37.4) | 458 (30.9) |  | 3,632 (24.2) | 316 (22.5) | 193 (19.0) |
| Parental body mass index, mean (SD), kg/m^2^ | 26.5 (4.8) | 26.9 (4.9) | 27.8 (5.2) |  | 27.2 (3.8) | 27.8 (3.9) | 28.6 (4.1) |

^a^ ≥12 years of education
