# Supplementary material for: Parental opioid prescriptions and the risk of opioid use in adolescents and young adults: The HUNT Study linked with prescription registry data
Source: PLoS Med. 2025 Oct 23;22(10):e1004763. doi: 10.1371/journal.pmed.1004763 (PMC12548922; doi:10.1371/journal.pmed.1004763)
Supplement: S1 Fig — Variables in black boxes are confounders that are adjusted for in the analyses. To avoid potential residual confounding by offspring age, this variable was also used as a confounder in the analyses. Abbreviations: MSK, musculoskeletal; BMI, body mass index; SES, socioeconomic status. (DOCX) [file pmed.1004763.s009.docx]

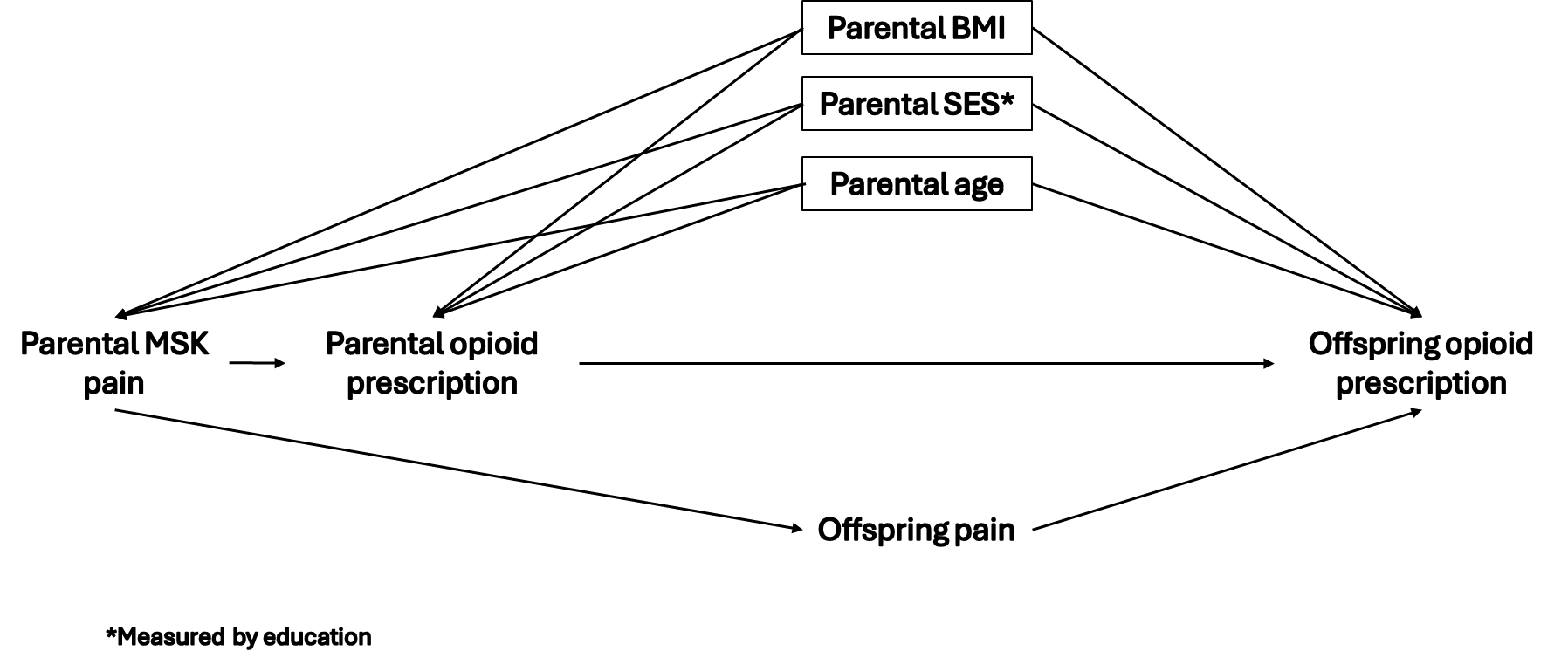


Figure S1. Direct acyclic graph. Variables in black boxes are confounders that are adjusted for in the analyses. To avoid potential residual confounding by offspring age, this variable was also used as confounder in the analyses. Abbreviations: MSK: musculoskeletal; BMI, Body mass index; SES: Socioeconomic status.
