# Supplementary material for: Parental opioid prescriptions and the risk of opioid use in adolescents and young adults: The HUNT Study linked with prescription registry data
Source: PLoS Med. 2025 Oct 23;22(10):e1004763. doi: 10.1371/journal.pmed.1004763 (PMC12548922; doi:10.1371/journal.pmed.1004763)
Supplement: Attachment — Submitted filename: Response_toReviewers_ToSubmit.docx [file pmed.1004763.s013.docx]

**POINT-BY-POINT RESPONSE TO REVIEWERS**

**Manuscript title:** Parental opioid prescriptions and the risk of opioid use in adolescents and young adults: The HUNT Study linked with prescription registry data

**Responses to editor and reviewers’ comments:**
We thank the editor and reviewers for the positive evaluation. The manuscript has been revised in accordance with the comments and suggestions.

**REVIEWER #1**

Marcuzzi and team utilize a large cohorts and data linkage to explore the associations between parental opioid use and subsequent offspring opioid use. This is an important question that may offer insights into targeted interventions for reducing opioid use in the community.

Main queries for authors:
1.     How were the linkages performed? Was this via unique identifying number and parent/child identifier or probabilistic matching? If the latter, what were the proportion of successful linkages?

**Response**: The linkages were established using the unique personal identification number assigned to all Norwegian citizens. This enables complete matching of offspring and parents participating in the HUNT Study and ensures individual-level linkage to the prescription registry data, independent of other family members. This has been added in the revised manuscript (Methods, study sample section, page 6):

*“Each participants record in the HUNT Study was linked via unique personal identification number with…”*

2.     Given a Cox model was used was the proportional hazard assumption checked? This should be included.

**Response:** The proportional hazard assumption was assessed using both graphical methods (log-log plots) and test of Schoenfeld residuals, and no violations were observed for any of the variables in the model. This has now been described in the revised manuscript (Methods, statistical analysis section, page 9).

*“Proportional hazard assumptions were assessed both graphically (log-log plots) and by test of Schoenfeld residuals, and no violations were observed.”*

3.     95% confidence interval are missing for all crude values in the tables, these must be included.

**Response:** We have now included the 95% confidence interval for the crude estimates in Table 2 and 3 and supplementary tables.

4.     The authors have assumed that those without the reimbursement code for opioid use were for non-malignant pain and were included. Is this assumption based on anything in terms of reimbursement reporting? How many had a missing reimbursement code? This is a significant assumption that may alter the included population.

**Response:** We understand that this can be confusing. The prescription database (NorPD) does not include prescription indications; however, the reimbursement codes (based on ICD10/ICPC-2) can in some circumstances serve as a proxy for diagnoses. These codes are issued for chronic conditions requiring long-term medication (e.g., asthma, cardiometabolic conditions, and cancer). Thus, not all prescriptions have a reimbursement code. For the scope of this study, identifying the specific indication for opioid prescription was not essential, providing that prescriptions due to malignancy (where dispensing opioids is indicated for pain relief) were identified and excluded. To avoid ambiguity, we removed the sentence referring to this (i.e., reimbursement codes indicating severe chronic pain (-71) or prescriptions with no reimbursement code) as this information is redundant given our explicit exclusion of malignancy-related prescriptions (Methods, linkage to the prescription database, page 7).

5.     Why were those with missing BMI data excluded? Was imputation considered for this variable?

**Response:** We thank the reviewer for this observation and agree that multiple imputation can be useful when including BMI as a confounder in the analysis. Thus, we have now performed multiple imputation for parental BMI and re-estimated all results, updated Table 2 and 3, as well as Figure 1. A description of the imputation method is included in the Methods section (other variables section, page 9).

We also noticed a small error as there were 23 participants with missing data on parental education. Therefore, this variable was also imputed (also described in Methods, other variables section, page 9).

*“Body mass index in mothers (n=423) and fathers (n=373), and parental education (n=23) had missing data that were imputed (20 imputations) using all variables in the main analysis as predictors in the imputation model.”*

6.     It is likely that those with missing information on pain may not be missing at random and therefore excluding these cases may introduce bias. The authors should consider imputation or sensitivity analyses exploring this.

**Response:** We understand the concern of the reviewer and acknowledge that selection based on having data on pain may introduce bias. However, we are reluctant to conduct multiple imputation analysis on this variable. First, stratifying on an imputed variable is not straight forward, since the stratum classification variable (i.e. pain or not) will vary for each imputed data set. Moreover, if the argument for doing multiple imputation is that missing is not at random, this violates one of the main assumptions for this approach. We would therefore prefer to not use imputation methods for this variable.

However, we have examined possible differences in parental age and opioid prescriptions between those with and without missing values on pain (see table below). There were no large differences that are likely to influence the results substantially. These results have been included as a Supplementary (Table S8) and we commented on this in the Discussion (Strengths and limitations section, page 17). We hope this is satisfactory.

*“Missing data on chronic MSK pain in parents was not imputed since this variable was used as a stratum classification variable. Although this may have contributed to selection bias, neither parental age nor opioid prescriptions were substantially different between those with and without data on parental MSK pain (supplementary Table S8).”*

|  | Not missing pain | Missing pain |
| --- | --- | --- |
| Mother age, mean (SD) | 46.4 (6.2) | 43.9 (6.8) |
| Mother opioid prescriptions, n (%)  0  1  ≥2 | 15,413 (84.0)  1,559 (8.5)  1,367 (7.5) | 1,657 (86.6)  124 (6.5)  132 (6.9) |
| Father age, mean (SD) | 49.7 (6.7) | 47.9 (7.5) |
| Father opioid prescriptions, n (%)  0  1  ≥2 | 13,432 (85.8)  1,283 (8.2)  935 (6.0) | 2,247 (90.5)  125 (5.0)  110 (4.4) |

7.     The persistent use and number of prescriptions should be consistent, it is confusing to switch between >3/4 of the year and >2 prescriptions.

**Response:** We thank the reviewer for pointing this out. We have chosen two different methods due to relatively small number of parents having prescriptions in >3/4 of the year. Specifically, for the persistent opioid prescription outcome there were only 21 offspring cases (vs 42 when using ≥2 prescriptions) in analysis of the mothers’ opioid use, and 11 vs 26 offspring cases for the corresponding analysis of fathers’ opioid use. We would also prefer to keep the offspring outcome definition since it more clearly reflects persistent use as defined in previous literature. We have rephrased this in the limitation section of the revised manuscript (Discussion, limitations section, page 17):

*“We did not categorise parental persistent opioid use with the same definition as offspring opioid use due to few cases and lack of statistical power.”*

8.     Adjusted analyses: How were covariates selected? Were direct acyclic graphs used? If so, please include this.

**Response:** We have made a DAG before carrying out the analyses and now added it to this submission in the supplementary file (Figure S1). The reference to the supplementary file was added to the revised manuscript (Methods, Other Variables, page 8).

Figure S1. Direct acyclic graph. Variables in black boxes are confounders that are adjusted for in the analyses. Examples of possible indirect effects are also indicated in boxes with dashed lines. To avoid potential residual confounding by offspring age, this variable was also used as confounder in the analyses. Abbreviations: BMI, Body mass index; SES: Socioeconomic status.

9.     Were other variables of socioeconomic position considered such as postcode to capture more deprived areas, household wealth, alcohol and substance use etc. Given the interrelatedness of SES and opioid use/misuse fully capturing this is critical, it is plausible that familial deprivation is driving this association.

**Response:** We agree that SES could be an important factor in these analyses. Postcode is rarely used as an indicator of deprived areas in a Norwegian context as it is unlikely to capture important SES variation, especially in this relatively rural area. Unfortunately, we did not have information on income in our data, but we believe that the high-quality registry-based data on education captures important variations in SES. Moreover, the negative control analysis was mainly included as an attempt to assess the potential SES confounding issue. The rational for this analysis is that a causal effect of parental opioid prescription on offspring BMI is unlikely, but it is likely that the confounding structure is similar to that of the parent-offspring opioid prescription association, in particular for SES. We have elaborated on this limitation in the revised manuscript (Discussion, limitations section, page 18):

*“Controlling for differences in socioeconomic status might be of particular importance, but indicators such as income or area-based socioeconomic level were either not available or not highly relevant in this homogenous and relatively rural setting. However, the complete registry-based education history available in this study is likely to capture important variation in socioeconomic status in the Norwegian context. Moreover, the negative control outcome analysis did not indicate residual confounding by such factors.”*

10.    Why was parental BMI selected and not offspring BMI? BMI has been associated with opioid use, thus this should be focused on the child. Is this not available and parental BMI a proxy for this? If so this should be stated.

**Response:** Although we have information on both parental and offspring BMI, we chose to use parental BMI in the regression models under the assumption that parental BMI is more likely to causally influence the exposure (i.e. parental opioid prescription) than offspring BMI, and can also influence offspring opioid prescription, for example through offspring BMI (see DAG above). We have also added a sentence in the revised manuscript to explain this (Methods, Other Variables section, page 8):

*“We controlled for parental body mass rather than offspring body mass as this factor is more likely related to both the exposure (i.e., parental opioid prescription) and the outcome (offspring opioid prescription), the latter through, for example, offspring body mass index.”*

11.     For the increase in opioid use with offspring age, was this statistically different? If not, this should be included. Additionally, caution is needed when interpreting this result, this will likely in some part be due to provider preference to avoid opioids in minors, especially for persistent use.

**Response:** We agree with the reviewer that avoiding multiple opioid prescriptions in adolescents might be one of the reasons for the opioid use increasing with age. We have added this point in the discussion (implications section, page 16):

*“It is possible that this increase can be related to more restrictive prescription practices in younger adolescents.”*

We have also assessed a possible linear trend in opioid prescription rates across age (described in Methods, Statistical Analysis section, page 9), and reported the results in the Results section (page 11):

*“Linear trends in opioid prescription rates were estimated from least-squares linear regression across the age groups and weighted by the inverse variance of the incidence rate.”*

*“This corresponded to a 4.9% (95% CI -0.6 to 10.4, p=0.06) increase for each five-year age group.”*

*“…corresponding to a 12.1% (95% 3.8 to 20.4 p=0.02) increase for each five-year age group.”*

12.    Age </>20 does not seem the best surrogate for living in the home, especially in setting with complex families (opioid use and chronic pain) and to me is insufficient to suggest that 'contextual factors' might be less relevant (line 248).

**Response:** We agree with the reviewer that the dichotomised age variable is insufficient to suggest that contextual factors might be less relevant, and we acknowledge that different family structures are likely to exist especially among those with more complex problems. We have amended this in the discussion (page 15).

*“Our analysis stratified by offspring age (i.e., <20 years, ≥20 years) did not show any substantial difference in the estimates between age groups, suggesting that living in the same household with parents did not have a differential effect on the risk of opioid prescription in offspring. However, offspring age may not fully capture family living conditions, both in terms of when offspring move out of the family home and due to more complex family structures, particularly in families experiencing greater burden (e.g., chronic pain and opioid use).”*

13.    BMI does not present as a negative control for me; BMI is associated with opioid use and offspring and parental BMI are linked. Unsure whether this analysis is additive to the manuscript.

**Response:** We understand the concern of the reviewer. However, as described in the response to comment 9 above, we think that the negative control analysis could contribute to assess the potential influence of SES on the estimated associations. As such, we would like to keep it as supplementary analysis.

Minor:
1.     Table 1: suggest making simpler with no opioid vs any opioid use and putting the stratified into the supps

**Response:** We thank the reviewer for this suggestion. We changed Table 1 accordingly and moved the original Table 1 to the supplementary material (Table S9).

2.     Suggest toning down of language -line 235, rather was associated with?

**Response:** We have changed the wording “strongly related to” to “associated with” (discussion, page 14):

*“We found that opioid prescription in parents was associated with an increased risk of persistent opioid use in their offspring.”*

3.     Line 74 - secondary aim could be removed from introduction

**Response:** We agree, and we have now removed the secondary aim from both the introduction (page 6) and the abstract (page 2).

4.     Line 61 simpler to say confounding by indication? And be clarified throughout that pain was included to investigate confounding by indication.

**Response:** We agree that offsprings’ possible higher susceptibility to pain due to parental pain, and thus possible higher likelihood of opioid use, could be labelled confounding by indication. However, this is a technical term that we think could confuse the readers, and we would like to keep the original phrasing in the introduction. However, we have clarified that this falls under the concept of confounding by indication in the Methods sections (page 9):

*“To address potential confounding by indication caused by increased susceptibility to pain in offspring of parents with chronic MSK pain, the association between parental opioid prescription (no prescription vs ≥2 prescriptions) and risk of any opioid prescription in offspring was analysed both as a joint variable with parental chronic MSK pain (no/yes) as well as stratified according to parental MSK pain.”*

5.     Psychological challenges - line 52 to be clarified

**Response:** We have further clarified this in the Introduction (page 5):

*“…psychological challenges, e.g., mood disorders, substance abuse, childhood trauma.”*

**REVIEWER #2**

This paper addresses an important topic. Strengths include linkage with the Norway prescription drug database; however, the low participation in the Hunt study (~54%) raise concerns about generalizability and potential selection bias.

1. My main concern for this study is the potential for selection bias. For example, there may be immortal time bias as parents may fill a prescription on some date but the survey is completed sometime later - and yet follow up starts not at the time of the prescription fill but at survey completion, i.e., the offspring are "immortal" between the exposure (prescription) and survey completion.

**Response:** We thank the reviewer for pointing this out. We use survey data as start of follow-up for the offspring since registry data can only be obtained among survey participants. Thus, we believe selection bias could potentially be stronger if prescriptions occurring before participation were included in the incidence rate. While we acknowledge that parental and offspring opioid use are not necessarily temporally aligned, our aim was not to demonstrate such an immediate effect. Rather, we view parental opioid prescriptions at any timepoint as indicative of a broader liability to opioid use that may be transferred to their offspring.

Also, as suggested in comment 3 below, to minimise the influence of prevalent opioid use, we conducted a sensitivity analysis excluding offspring with opioid prescriptions one year prior to survey participation and found similar results.

Additionally, participation in the Hunt study is very low. What is the population that is represented by the participants in this study? How generalizable are the findings?

**Response:** Although a representative sample is important for descriptive inferences to the wider population, it has been argued the this is not the case for studies aiming at estimating valid exposure-outcome associations, such as in the current study (Rothman et al (2013), *Why representatives should be avoided*, <https://doi.org/10.1093/ije/dys223>). We therefore believe that although close to 50 percent of the invited did not participate in the HUNT Study, the results can still be generalized to a wider population under the assumption of high internal validity (i.e., not strongly biased results).

2. I'm unclear why parental BMI is a confounder in this study. For a variable to be a confounder, it must be a common cause of the exposure and outcome. How is parental BMI a cause of opioid prescriptions in offspring?

**Response:** We think it is possible that parental BMI would influence offspring opioid prescription through various mechanisms, including through offspring BMI. In our response to Reviewer 1 (comment 8), we have included a directed acyclic graph (DAG) displaying these effects in the supplementary file of the revised manuscript (Figure S1). We have also added a sentence in the revised manuscript to describe this (Methods, Other Variables section, page 8):

*“We controlled for parental body mass rather than offspring body mass as this factor is more likely related to both the exposure (i.e., parental opioid prescription) and the outcome (offspring opioid prescription), the latter through, for example, offspring body mass index.”*

3. The authors raise the issue of offspring opioid use outside the time window. Why not apply a washout period (e.g., 6 months - 1 year) to ensure that the offspring in your sample had no prior prescriptions?

**Response:** We thank the reviewer for this suggestion. We have rerun the analysis excluding those that had opioid prescription 1 year prior to participation (n=739) and reported the results in supplementary analyses (Table S5). No substantial differences were observed between the estimates for this analysis and the main analysis. We have added this to the revised manuscript (Methods, statistical analysis section, page 10; Results, page 14; Discussion, limitation section, page 17):

*“Also, we excluded offspring with any opioid prescription one year prior to the survey participation (i.e., start of follow up) to avoid influence of prevalent opioid use.”*

*“In supplementary Table S5, the HRs of opioid prescriptions in offspring were similar to those of the main analysis, after excluding offspring who had an opioid prescription one year prior to survey participation.”*

*“It is also possible that some offspring had opioid prescriptions before their entry into the study, and this might have resulted in underestimated associations. However, we conducted a sensitivity analysis excluding offspring with opioid prescriptions one year prior to study participation, and the results remained similar to the main analyses.”*

4. It seems that there is missing data with respect to the reimbursement code for some prescriptions. What is the prevalence of missingness? The ad hoc method to classify "prescriptions with no reimbursement code … as prescriptions due to non-malignant pain" can introduce bias depending on the degree of missingness.

**Response:** We thank the reviewer for this comment. We have addressed this point already in response to Reviewer 1 (comment 4) as follow:

“The prescription database (NorPD) does not include prescription indications; however, the reimbursement codes (based on ICD10/ICPC-2) can in some circumstances serve as a proxy for diagnoses. These codes are issued for chronic conditions requiring long-term medication (e.g., asthma, cardiometabolic conditions, and cancer). Thus, not all prescriptions have a reimbursement code. For the scope of this study, identifying the specific indication for opioid prescription was not essential, providing that prescriptions due to malignancy (where dispensing opioids is indicated for pain relief) were identified and excluded. To avoid ambiguity, we removed the sentence referring to this (i.e., reimbursement codes indicating severe chronic pain (-71) or prescriptions with no reimbursement code) as this information is redundant given our explicit exclusion of malignancy-related prescriptions (Methods, linkage to the prescription database, page 7).”

5. There are some illogical statements in the Discussion. For example, the authors state "Thus, the strong association between parent and offspring opioid prescription observed in this study highlights the need for increased awareness among health personnel that familial factors are important to consider when prescribing opioids as part of managing pain conditions in adolescents and young adults." But this study looked at prescribing opioids to parents, so this conclusion is not supported.

**Response:** We understand that this statement might generate misunderstanding, but we believe that this is a fair interpretation of the results as parental opioid is the exposure, and the target population of the study is adolescents and young adults. Thus, what we are trying to convey with this statement is that health care personnel handling chronic pain in this patient group should be aware that opioid use in parents is a risk factor for own (offspring) opioid use.

**REVIEWER #3**

A useful contribution to an accumulating body of evidence about family (parental) prescription opioid use and its interesting connection with offspring prescription opioid use. The study usefully builds upon prior evidence in several ways. First, the predictors and outcome are conceptualized as receipt of prescriptions for an opioid compound -- not harmful or hazardous use, and possibly not even use, to the extent that some opioid prescriptions are issued/filled but then the compound is not taken. Second, there is a clearly specified epidemiologically credible population under study with a well-described sample of sufficient size to yield reasonably precise hazard ratio estimates. Third, the study design is prospective, whereas most evidence is based on non-prospective designs. Fourth, the analysis and estimation approaches are described with clarity.

The authors have an opportunity to say more about potential public health tactics and what clinicians might do in office-based practices. It is not clear that many readers will be surprised to find that there is some degree of within-family interdependence of medicine-seeking outcomes, although the focus on prescription-only opioids use is relatively novel (i.e., in contrast with the many studies of opioids used outside of the boundaries of clinically prescribed instructions).

Some value might be gained by considering the early sociological studies of diffusion of prescribing practices -- i.e., interdependencies within practice settings -- and work in the direction of treatment choices. This body of evidence generally focuses upon the behavior of clinician-prescribers and the choices they make on behalf of their patients. The conceptual models that guided these studies of clinicians might prove to offer guidance for conceptual models needed to interpret this study's interesting estimates. At first blush, the mechanism of behavioral imitation might come to mind, but the choice models and discussions of these types of interdependencies offer more intriguing possibilities (e.g., see Manski on treatment choice and the difficulties faced when distinguishing what might be called 'social effects' from other sources of interdependencies).

**Response:** We appreciate these valuable suggestions from the reviewer and looked further into the literature to expand more on the mechanisms and the implication (as suggested in the first part of the reviewer’s comment). The following changes were made to the Discussion section (page 15 and implications section in page 16) which we hope are satisfactory:

*“Several mechanisms may mediate this association, including parental norms and preferences regarding the use of analgesics (30), shared health-seeking behaviour influenced by familial vulnerability to illnesses, role modelling, and broader family context (31). Similarly, clinician preferences in prescribing practices may also contribute to this association (16, 17), particularly in the Norwegian context, where it is common that offspring and parents share the same general practitioner (32). Variations in clinical practice may also influence preferences towards a particular general practitioner which could further reinforce this association (31).”*

In implications:

*“For example, history of familial opioid prescription could help identify patients with particularly high risk of prolonged opioid use in a clinical setting. From a public health perspective, the value of family-based preventive strategies, and, more broadly, multilevel interventions (e.g., targeting both the family and the clinicians) should be explored to increase efforts in preventing potentially unnecessary opioid use in young people (41). This would also require a better understanding of processes underlying social interactions, e.g., among family members, friends, health care personnel, and their influence on individual patients’ decision-making (42).”*

Some word choices might be re-considered. For example, the abstract starts by seeming to promise causal inference, but the reader will be disappointed if the expectation is definitive evidence strong enough to support causal inference.

**Response:** We agree with the reviewer, and we have revised the abstract (page 2) and the introduction (page 5 and 6) throughout to avoid a strong causal language.

**GENERAL EDITORIAL REQUESTS**

We ask every co-author listed on the manuscript to fill in a contributing author statement, making sure to declare all competing interests. If any of the co-authors have not filled in the statement, we will remind them to do so when the paper is revised. If all statements are not completed in a timely fashion this could hold up the re-review process. If new competing interests are declared later in the revision process, this may also hold up the submission. Should there be a problem getting one of your co-authors to fill in a statement we will be in contact. Please do not add or remove authors without first discussing this with the handling editor. You can see our competing interests policy here:
<http://journals.plos.org/plosmedicine/s/competing-interests>.

*      Please upload any figures associated with your paper as individual TIF or EPS files with 300dpi resolution at resubmission; please read our figure guidelines for more information on our requirements: <http://journals.plos.org/plosmedicine/s/figures>. While revising your submission, please upload your figure files to the PACE digital diagnostic tool, <https://pacev2.apexcovantage.com/>. PACE helps ensure that figures meet PLOS requirements. To use PACE, you must first register as a user. Then, login and navigate to the UPLOAD tab, where you will find detailed instructions on how to use the tool. If you encounter any issues or have any questions when using PACE, please email us at [PLOSMedicine@plos.org](mailto:PLOSMedicine@plos.org).

**Response:** This was done.

*      Please ensure that the paper adheres to the PLOS Data Availability Policy (see <http://journals.plos.org/plosmedicine/s/data-availability)>, which requires that all data underlying the study's findings be provided in a repository or as Supporting Information. For data residing with a third party, authors are required to provide instructions with contact information (web or email address) for obtaining the data. Please note that a study author cannot be the contact person for the data. PLOS journals do not allow statements supported by "data not shown" or "unpublished results." For such statements, authors must provide supporting data or cite public sources that include it.

**Response:** A data availability statement was provided.

*      We expect all researchers with submissions to PLOS in which author-generated code underpins the findings in the manuscript to make all author-generated code available without restrictions upon publication of the work. In cases where code is central to the manuscript, we may require the code to be made available as a condition of publication. Authors are responsible for ensuring that the code is reusable and well documented. Please make any custom code available, either as part of your data deposition or as a supplementary file. Please add a sentence to your data availability statement regarding any code used in the study, e.g. "The code used in the analysis is available from Github [URL] and archived in Zenodo [DOI link]" Please review our guidelines at <https://journals.plos.org/plosmedicine/s/materials-software-and-code-sharing> and ensure that your code is shared in a way that follows best practice and facilitates reproducibility and reuse. Because Github depositions can be readily changed or deleted, we encourage you to make a permanent DOI'd copy (e.g. in Zenodo) and provide the URL.

**Response:** Not applicable.

*      Please ensure that the study is reported according to the STROBE guideline and include the completed STROBE checklist as Supporting Information. When completing the checklist, please use section and paragraph numbers, rather than page numbers. Please add the following statement, or similar, to the Methods: "This study is reported as per STROBE guideline (S1 Checklist)."

**Response:** A STROBE checklist was added to the submission together with the statement.

FORMATTING – GENERAL

*      At this stage, we ask that you include a short, non-technical Author Summary of your research to make findings accessible to a wide audience that includes both scientists and non-scientists. The Author Summary should immediately follow the Abstract in your revised manuscript. This text is subject to editorial change and should be distinct from the scientific abstract. Ideally each sub-heading should contain 2-3 single sentence, concise bullet points containing the most salient points from your study. In the final bullet point of 'What Do These Findings Mean?', please include the main limitations of the study in non-technical language. Please see our author guidelines for more information:
<https://journals.plos.org/plosmedicine/s/revising-your-manuscript#loc-author-summary>.

**Response:** This was included.

*      Please express the main results with 95% CIs as well as p values. When reporting p values please report as p<0.001 and where higher as the exact p value p=0.002, for example. Throughout, suggest reporting statistical information as follows to improve clarity for the reader "22% (95% CI [13%,28%]; p</=)". Please be sure to define all numerical values at first use.

**Response:** In line with the current recommendations of the American Statistical Association (<https://www.amstat.org/asa/files/pdfs/p-valuestatement.pdf>) and others, we are reluctant do report p-values in addition to the confidence intervals. The 95% confidence interval conveys all information about precision of the estimated associations, and at the same time avoids a dichotomised classification of results into those that are significant or not.

SUPPLEMENTARY MATERIAL
*      Please note that supplementary material will be posted as supplied by the authors. Therefore, please amend it according to the relevant comments outlined here.

*      Please cite your Supporting Information as outlined here: <https://journals.plos.org/plosmedicine/s/supporting-information>

**Response:** This was done.

REFERENCES
*      PLOS uses the numbered citation (citation-sequence) method and first six authors, et al.

*      Please ensure that journal name abbreviations match those found in the National Center for Biotechnology Information (NCBI) databases (<http://www.ncbi.nlm.nih.gov/nlmcatalog/journals>), and are appropriately formatted and capitalised.

*      Where website addresses are cited, please include the complete URL and specify the date of access (e.g. [accessed: 12/06/2023]).

*      Please also see <https://journals.plos.org/plosmedicine/s/submission->guidelines#loc-references for further details on reference formatting.

**Response**: This was done.

[STUDY TYPE-SPECIFIC REQUESTS - DELETE SECTIONS AS NECESSARY]
OBSERVATIONAL STUDIES

*      Abstract: Please include the study design, population and setting, number of participants, years during which the study took place (enrollment and follow up), length of follow up, and main outcome measures.

**Response**: The years during which the study took place was added to the abstract.
