# Supplementary material for: Parental opioid prescriptions and the risk of opioid use in adolescents and young adults: The HUNT Study linked with prescription registry data
Source: PLoS Med. 2025 Oct 23;22(10):e1004763. doi: 10.1371/journal.pmed.1004763 (PMC12548922; doi:10.1371/journal.pmed.1004763)
Supplement: Attachment — Submitted filename: Response_toReviewers_R2_ToSubmit.docx [file pmed.1004763.s014.docx]

**POINT-BY-POINT RESPONSE TO REVIEWERS**

**Manuscript title:** Parental opioid prescriptions and the risk of opioid use in adolescents and young adults: The HUNT Study linked with prescription registry data

**Responses to editor and reviewers’ comments:**
We thank the editor and reviewers for the additional comments. We have revised the manuscript in accordance with the suggestions.

**REVIEWER #1**

I thank the authors for their considered replies. I have some remaining queries:

1. Further detail of follow-up is needed to ensure immortal time bias is not present and reverse causation. Parental opioid use after offspring participation (up to 3 years post - as per page 7 of methods) has a high-level potential for immortal time bias and reverse causation (exposure measured after the outcome). Please clarify.

**Response**: We understand the concerns of the reviewer. The reason for our approach was to capture parental liability to opioid use, and not necessarily the direct temporal effect. This has been further clarified in the Introduction of the revised manuscript as follow (page 6):

*“We hypothesised that parental opioid prescriptions would be associated with an increased risk of offspring’s opioid prescriptions as an indication of familial predisposition to opioid use.”*

We don’t think that immortal time bias is present since all prescriptions in the offspring are counted from baseline. However, we are aware that some parental opioid prescriptions in the three-year period after start of follow-up could be related to preceding offspring opioid use. We have now included two sensitivity analyses: one including only parental opioid prescriptions before start of follow-up, and one including only parental opioid prescriptions after start of follow-up to assess the possible influence of the timing of exposure assessment. We have added this in the methods, statistical analysis (page 10) and results (page 14):

*“Finally, to assess the possible influence of the timing of exposure assessment, we conducted two sensitivity analyses: 1) including only parental opioid prescriptions in the 2-year period prior to the start of follow-up, and 2) including only parental opioid prescriptions in the 3-year period after the start of follow-up.”*

*“Finally, using only parental opioid prescriptions from either before or after start of follow-up gave largely similar HRs as in the main analysis (supplementary Table S8).”*

We have also expanded on this in the limitation section (page 17):

*“**In addition, parental opioid prescriptions were measured both before and after offspring HUNT participation. This may increase the likelihood that some parental opioid prescriptions in the tree-year period after start of follow-up could be related to preceding offspring opioid use (i.e., reverse causation).* *However, sensitivity analyses using only parental opioid prescriptions either before or after start of follow-up yielded largely similar estimates as the main results using the 5-year period.”*

2. Recognition the reimbursement codes were not available for all prescriptions as a limitation.

**Response**: We thank the reviewer for this remark. However, reimbursements codes are only registered for “blue prescriptions” that are either partly or completely covered by the state for severe and long-term illness. Management of MSK conditions are often not approved for a blue prescription. Thus, we do not think that this is a limitation, as we did not classify opioid prescriptions based on the indication but only use reimbursement codes to exclude prescriptions related to malignant pain. We think that adding this to the limitation can be confusing, but have further clarified this in the Methods section (page 7):

*“Reimbursement codes are only issued for chronic conditions that require long-term medication (i.e., blue prescriptions) and thus, not all prescriptions have a reimbursement code.”*

3. Imputation of BMI - was the adequacy of the imputation checked and assumptions of mode of missing checked prior? (ie missing at random).

**Response**: The reason for missing data on BMI was mainly that participants did not take part in the clinical measurements due to e.g., travel distance, health status, or being institutionalised. It is therefore likely that the missingness is not completely at random but related to other factors (i.e., missing at random). Thus, it is important that there is compatibility between the analysis model and the imputation model. In our case, the imputation model was based on chained equations (*linear* for BMI and *logit* for higher education) using all factors in the analysis model as predictors. This has been further specified in the Methods section (page 9):

*“Body mass index in mothers (n=423) and fathers (n=373), and parental education (n=23) had missing data that were imputed (20 imputations) based on chained equations (linear for body mass index and logit for higher education) using all variables in the main analysis, including the outcome variable, as predictors in the imputation model.”*

4. Missing chronic pain - I agree that imputation would not be appropriate as it does not appear missing at random. Although the authors state there are no large differences this is not true: missing vs non missing for maternal opioid prescription; 1,559 (8.5%) vs 124 (6.5%) - Z test p-value; 0.018 and paternal 1,283 (8.2%) vs 125 (5.0%); p-value: <0.00001 and are likely not only statistically different but clinically meaningful. This should be noted in the results and discussion of this amended given the difference is not negligible.

**Response**: We agree that these proportions are somewhat different, and we have now stated this in the method, statistical analysis section (page 10):

“*A somewhat higher proportion of mothers and fathers had opioid prescriptions if they had missing data on chronic pain compared to those without missing data (supplementary table S7).”*

And added this to the discussion, limitation section (page 18):

*“While parental age was similar between those with and without data on parental MSK pain, the proportion with parental opioid prescription was somewhat larger in the latter group (supplementary Table S7). This may contribute to selection bias, thus underestimating the joint association of parental pain and parental opioid prescriptions with offspring opioid prescriptions”*

5. The DAG is incomplete and does not consider the associations between the covariates. The inclusion of eg and multiple covariates is confusing. Chronic pain should be its own variable as an ancestor of exposure. Noting adjustment of the available covariates; Parental age, Parental education, parental BMI does seem sufficient to close biasing paths. However residual confounding is plausible, especially given other substance use data unavailable and should be noted as a limitation.

**Response**: We thank the reviewer for this suggestion and have now modified the DAG accordingly (see below, also in supplementary figure S1).

We also agree that residual confounding might still be present, and this was already partly acknowledged in the limitation, but we added substance use as an unmeasured factor in the limitation (page 18):

*“Finally, we cannot rule out residual confounding due to unmeasured factors, e.g., family socioeconomic status, parental chronic diseases and other substance use influencing both their own and their offspring’s risk of opioid prescriptions.”*

6. I do not agree with the use of BMI as a negative control; it is a confounder and clearly so in the DAG. A negative control for exposure must be a variable that does not affect the outcome.

**Response**: We have removed the negative control analysis from the revised manuscript.

**REVIEWER #2**

Thank you for addressing my first set of comments. I appreciate the authors conducting a sensitivity analysis to address potential bias due to prevalent opioid use. However, some of the responses are inadequate or inaccurate.

For example, the idea that sample representativeness is not important in explanatory research is outdated. There is now robust literature that representativeness is important when there is treatment effect heterogeneity across sample characteristics, and those characteristics influence selection into the study sample. When both are true, estimates obtained in the selected sample will not generalize (i.e., transport) to the target population where clinical or policy decisions will be made (e.g., Hayes-Larson et al. 2024 Epidemiology). Additionally, the idea that results can be generalized to a wider population under the assumption of high internal validity is incorrect. A double-blind RCT with perfect adherence and no attrition would have high internal validity, but this does not guarantee its external validity (e.g., if trial participants are healthier, more motivated etc. than the general population.)

**Response**: We thank the reviewer for this thorough comment. We agree that internal validity in not sufficient to ensure generalisability, although it is a necessary requirement, and that there are other factors that influence the generalisability of the findings. While we acknowledge that the strength of the association may depend on the sample under study (i.e. selection bias), we believe that the findings can still be generalised to the wider Norwegian population and also to other populations with similar family and health care structures. We have expanded on this in the limitation section (page 17):

*“…it is possible that the study comprised a healthier sample compared to non-responders that could contribute to underestimate the parent-offspring associations of opioid use. Although the results from this study may be particularly relevant in a Norwegian setting, the findings may transport to other populations with similar family and health care structures.”*

I'm still unconvinced that parental BMI is an appropriate confounder to adjust for for this research question. While the new DAG is helpful, it would suggest from the authors' explanation that offspring BMI is a descendent of parental BMI. If the authors truly believe that offspring BMI is a strong risk factor or cause of opioid prescriptions in offspring, then offspring BMI seems like the appropriate measure to adjust for, since it is unlikely to be an instrument whereas parental BMI could be.

**Response**: Based on the first reviewer and editor comments, we would like to keep parental BMI as confounder. The rationale for including parental BMI was explained in the revised manuscript in the methods, other variables section as follow: “We controlled for parental body mass index rather than offspring body mass index as this factor is more likely related to both the exposure (i.e., parental opioid prescription) and the outcome (offspring opioid prescription), the latter through, for example, offspring body mass index”.

We have also revised the DAG for better clarity (in supplementary figure S1), and hope this is satisfactory.

Finally, the sentence in question in the Discussion is still poorly written. "…when prescribing opioids as part of managing pain conditions in adolescents and young adults" suggests to the reader that clinicians may need to be aware when "managing pain conditions [using opioids] in adolescents and young adults." Because the exposure was parental opioids, clinicians may need to be aware when using opioids to manage pain in parents.

**Response**: We understand the point of the reviewer and we have rephrased that sentence in the discussion removing the ‘prescribing opioids’ part, implications section (page 16):

*“…highlights the need for increased awareness among health personnel that parental opioid use is important to consider when managing pain conditions in adolescents and young adults.”*

And also added the parents as target in the conclusions (page 18):

*“These findings suggest that family-based strategies, targeting both parents and their offspring, should be considered when managing pain and reducing potentially unnecessary opioid use in young people.”*

EDITOR SUGGESTIONS

As you will see, the reviewers think the manuscript is improved but still have several remaining concerns. After discussing the paper with the editorial team and an academic editor with relevant expertise, we are inviting you to revise the paper to address these remaining comments. Please ensure that you address all the remaining concerns from the reviewers.

Specifically, we would like you to:

1) Address the comment from Reviewer #1 and ensure that the DAG is complete. You may also want to consider removing offspring BMI from the DAG.

**Response**: We have now modified the DAG according to Reviewer #1 suggestions and removed offspring BMI.

2) Remove the analysis using BMI as a negative control, as BMI is a confounder.

**Response**: This analysis was removed.

3) Consider whether the conclusions can be generalizable. If not, please state this as a limitation in the discussion.

**Response**: We have expanded the limitation section on this (page 17):

*“…it is possible that the study comprised a healthier sample compared to non-responders that could contribute to underestimate the parent-offspring associations of opioid use. Although the results from this study may be particularly relevant in a Norwegian setting, the findings may transport to other populations with similar family and health care structures.”*

4) We are ok with parental BMI as a confounder (comment R2), but please ensure the explanation for this is clearly described in the manuscript.

**Response**: We had the explanation already in the Methods of the previously revised manuscript stating the following: “We controlled for parental body mass index rather than offspring body mass index as this factor is more likely related to both the exposure (i.e., parental opioid prescription) and the outcome (offspring opioid prescription), the latter through, for example, offspring body mass index”, and hope this is satisfactory.

GENERAL EDITORIAL COMMENTS:

FORMATTING - GENERAL

* In the last sentence of the Abstract Methods and Findings section, please describe the main limitation(s) of the study's methodology.

**Response**: This was added to the abstract.

*Non-technical Author summary:

-Please consider adding a sentence about the analysis performed to the "What did the researchers do and find?" section.

**Response**: This was added.

FIGURES AND TABLES

*	Please define all abbreviations used in each figure/table (including those in Supporting Information files).

**Response**: This has been checked.

For all observational studies, in the manuscript text, please indicate: (1) the specific hypotheses you intended to test, (2) the analytical methods by which you planned to test them, (3) the analyses you actually performed, and (4) when reported analyses differ from those that were planned, transparent explanations for differences that affect the reliability of the study's results. If a reported analysis was performed based on an interesting but unanticipated pattern in the data, please be clear that the analysis was data driven.

Response: We have added a sentence about the hypothesis at the end of the introduction as follow (page 6):

*“We hypothesised that parental opioid prescriptions would be associated with an increased risk of offspring’s opioid prescriptions as an indication of familial predisposition to opioid use.”*

The other points were already included in the manuscript.
