# Supplementary material for: Parental opioid prescriptions and the risk of opioid use in adolescents and young adults: The HUNT Study linked with prescription registry data
Source: PLoS Med. 2025 Oct 23;22(10):e1004763. doi: 10.1371/journal.pmed.1004763 (PMC12548922; doi:10.1371/journal.pmed.1004763)
Supplement: Attachment — Submitted filename: Response_toReviewers_R3_ToSubmit.docx [file pmed.1004763.s015.docx]

**POINT-BY-POINT RESPONSE TO REVIEWERS**

**Manuscript title:** Parental opioid prescriptions and the risk of opioid use in adolescents and young adults: The HUNT Study linked with prescription registry data

**Responses to editor and reviewers’ comments:**
We thank the editor and reviewers for the additional comments. We have revised the manuscript in accordance with the suggestions.

**EDITORIAL COMMENTS**

*We appreciate that you added the sensitivity analysis in Table S8. However, we would like to ask you to include this table in the main text. Furthermore, we would like you to provide a discussion of this table and the associated study limitation.

**Response:** We have now added the sensitivity analysis to the main text and further commented on the results in the limitation section of the discussion (page 19) as follow:

*“[…] Sensitivity analyses separating parental opioid prescriptions occurring either before or after start of follow-up yielded largely similar estimates as the main results.* *Furthermore, familial liability to opioid use may exert its effect irrespective of the timing of opioid prescriptions.”*

*Please add the numbers of the supplemental tables in the methods, where you describe these sensitivity analyses.

**Response:** This was added.

*If this is available, please provide more participant characteristics, for both parents and offspring. Including but not limited to income, education (more detailed than just % to 12 years), and ethnicity.

**Response:** We don’t have information about income, but we added more detailed information on education. Information on ethnicity has not been collected in HUNT, however the population is ethnically homogenous with the majority being Caucasian (>90%).

*Please include, either in the text or as a supplemental table, information about the timing of parental vs. offspring prescriptions.

**Response:** We have provided this information in the methods section (page 8) as follow:

*“In the subsample where both offspring and parents had an opioid prescription, the median [25^th^ percentile, 75^th^ percentile] days from a parental opioid prescription to an offspring prescription was 868 [-153, 2,467] for mothers and 1,004 [-92, 2,557] for fathers.”*

*Clarify if there is any data on offspring opioid prescriptions at baseline.

**Response:** We only included prescriptions from the date of baseline participation and ongoing prescriptions were not included unless they were renewed at/after the date of baseline. This was added to the methods (page 8) as follow:

*“Prescriptions were included from the date of survey participation while ongoing prescriptions were not included unless they were renewed at or after the date of participation.”*

**GENERAL EDITORIAL REQUESTS**

* Please confirm that your title complies with to PLOS Medicine's style. Your title must be nondeclarative and not a question. It should begin with main concept if possible. "Effect of" should be used only if causality can be inferred, i.e., for an RCT. Please place the study design ("A randomized controlled trial," "A retrospective study," "A modelling study," etc.) in the subtitle (ie, after a colon).

**Response:** The title complies with the PLOS Medicine style.

* Please confirm that your abstract complies with our requirements, including format (three sections: Background, Methods and Findings, and Conclusions) and providing all the information relevant to this study type <https://journals.plos.org/plosmedicine/s/submission-guidelines#loc-abstract>

**Response:** The abstract complies with the PLOS Medicine style.

* Please ensure that the Introduction ends with a clear description of the study question or hypothesis.

**Response:** This was checked.

* Please ensure that all abbreviations are defined at first use throughout the text.

**Response:** This was checked.

* Please confirm that all numbers presented in the abstract are present and identical to numbers presented in the main manuscript text.

**Response:** This was checked.

**GENERAL**

* Please remove the 'conclusions' subheading from the discussion. Please also remove any other subheadings from the discussion.

**Response:** This was done.

* Statistical reporting: Please revise throughout the manuscript, including tables and figures.

- Please report statistical information as follows to improve clarity for the reader ""22% (95% CI [13,28]; p</=)"".

- Please separate upper and lower bounds with commas instead of hyphens as the latter can be confused with reporting of negative values.

- Please repeat statistical definitions (HR, CI etc.) for each set of parentheses.

**Response:** The statistical reporting was changed to comply with the PLOS Medicine style.

* In the last sentence of the Abstract Methods and Findings section, please describe the main limitation(s) of the study's methodology.

**Response:** This was already included.

* In the abstract, please include the important dependent variables that are adjusted for in the analyses.

**Response:** This was added.

**FUNDING STATEMENT**

* The funding statement should include: specific grant numbers, initials of authors who received each award, URLs to sponsors’ websites. Also, please state whether any sponsors or funders (other than the named authors) played any role in study design, data collection and analysis, the decision to publish, or preparation of the manuscript. If they had no role in the research, include this sentence: “The funders had no role in study design, data collection and analysis, decision to publish, or preparation of the manuscript.

COMPETING INTERESTS STATEMENT

* All authors must declare their relevant competing interests per the PLOS policy, which can be seen here: <https://journals.plos.org/plosmedicine/s/competing-interests> For authors with ties to industry, please indicate whether any of the interests has a financial stake in the results of the current study.

**Response**: No conflict of interest to declare.

FIGURES

* Please show graph axes beginning at zero. If this is not possible, please show a break in the axis.

**Response:** We have now changed the graph y-axes of figure 2.

OBSERVATIONAL, COHORT, CROSS-SECTIONAL, AND CASE CONTROL STUDIES

* Did your study have a prospective protocol or analysis plan? Please state this (either way) early in the Methods section.

**Response**: A prospective protocol was not published, but the study was prospectively registered in OSF as part of a larger project as indicated in the methods.

a) If a prospective analysis plan (from your funding proposal, IRB or other ethics committee submission, study protocol, or other planning document written before analyzing the data) was used in designing the study, please include the relevant prospectively written document with your revised manuscript as a Supporting Information file to be published alongside your study, and cite it in the Methods section. A legend for this file should be included at the end of your manuscript.

b) If no such document exists, please make sure that the Methods section transparently describes when analyses were planned, and when/why any data-driven changes to analyses took place.

c) In either case, changes in the analysis-- including those made in response to peer review comments-- should be identified as such in the Methods section of the paper, with rationale.

**Response:** This was added in the statistical analysis section of the methods (page 11) as follow:

*“All main and supplementary analyses were pre-planned, with the exception of those presented in Table 4 and Table S7 which were included after peer-review.”*

* For all observational studies, in the manuscript text, please indicate: (1) the specific hypotheses you intended to test, (2) the analytical methods by which you planned to test them, (3) the analyses you actually performed, and (4) when reported analyses differ from those that were planned, transparent explanations for differences that affect the reliability of the study's results. If a reported analysis was performed based on an interesting but unanticipated pattern in the data, please be clear that the analysis was data-driven.

**Response:** This is described.

Comments from Reviewers:

**Reviewer #1**: I thank the authors for their considered replies and believe they have addressed most issues.

However, the concerns around reverse causation remain. If the editors are happy to accept parental opioid use without consideration to the temporal relationship with offspring use then I will not belabor the point.

Otherwise, I suggest removing those exposures that occurred after outcome. The aim of the study is to 'examine the association between parental opioid prescriptions and risk of opioid use in young people'. This cannot be assessed using an exposure (parental use) that captured after the outcome (child use). Supplemental Table 8 not address the concerns of reverse causation.

Additionally, the qualifier 'somewhat' on pages 10 and 18.

**Response**: We have now expanded on the limitation section of the discussion in line with the editor’s suggestion.

We removed somewhat from the two sentences identified by the reviewer.

**Reviewer #2**: The authors have addressed my comments.
